# Genomic Evaluation of Primiparous High-Producing Dairy Cows: Inbreeding Effects on Genotypic and Phenotypic Production–Reproductive Traits

**DOI:** 10.3390/ani10091704

**Published:** 2020-09-21

**Authors:** Miguel A. Gutiérrez-Reinoso, Pedro Manuel Aponte, Joel Cabezas, Lleretny Rodriguez-Alvarez, Manuel Garcia-Herreros

**Affiliations:** 1 Departamento de Ciencia Animal, Laboratorio de Biotecnología Animal, Facultad de Ciencias Veterinarias, Universidad de Concepción (UdeC), Chillán 3780000, Chile; mgutierrezreinoso@hotmail.com (M.A.G.-R.); joelcabezas@udec.cl (J.C.); 2 Facultad de Ciencias Agropecuarias y Recursos Naturales, Carrera de Medicina Veterinaria, Universidad Técnica de Cotopaxi (UTC), Latacunga 050150, Ecuador; 3 Colegio de Ciencias Biológicas y Ambientales (COCIBA), Universidad San Francisco de Quito (USFQ), Quito 170157, Ecuador; pmaponte@usfq.edu.ec; 4 Instituto de Investigaciones en Biomedicina “One-health”, Universidad San Francisco de Quito (USFQ), Campus Cumbayá, Quito 170157, Ecuador; 5 Instituto Nacional de Investigação Agrária e Veterinária (INIAV), 2005-048 Santarém, Portugal

**Keywords:** inbreeding index, homozygosis, genomic analysis, genotypic parameters, phenotypic traits, production, reproductive performance, primiparous, dairy cattle

## Abstract

**Simple Summary:**

Improving the genomic prediction methodologies in high-producing dairy cattle is a key factor for the selection of suitable individuals to ensure better productivity. However, the most advanced prediction tools based on genotyping show ~75% reliability. Nowadays, the incorporation of new indices to genomic prediction methods, such as the Inbreeding Index (II), can significantly facilitate the selection of reliable production and reproductive traits for progeny selection. Thus, the objective of this study was to determine the impact of II (low: LI and high: HI), based on genomic analysis, and its effect on production and reproductive phenotypic traits in high-producing primiparous dairy cows. Individuals with II between ≥2.5 and ≤5.0 have shown up to a two-fold increase in negative correlations comparing LI versus HI genomic production and reproductive parameters, severely affecting important traits such as Milk Production at 305 d, Protein Production at 305 d, Fertility Index, and Daughter Pregnancy Rate. Therefore, high-producing dairy cows face an increased risk of negative II-derived effects in their selection programs, particularly at II ≥ 2.5.

**Abstract:**

The main objective of this study was to analyze the effects of the inbreeding degree in high-producing primiparous dairy cows genotypically and phenotypically evaluated and its impacts on production and reproductive parameters. Eighty Holstein–Friesian primiparous cows (age: ~26 months; ~450 kg body weight) were previously genomically analyzed to determine the Inbreeding Index (II) and were divided into two groups: low inbreeding group (LI: <2.5; *n* = 40) and high inbreeding group (HI: ≥2.5 and ≤5.0; *n* = 40). Genomic determinations of production and reproductive parameters (14 in total), together with analyses of production (12) and reproductive (11) phenotypic parameters (23 in total) were carried out. Statistically significant differences were obtained between groups concerning the genomic parameters of Milk Production at 305 d and Protein Production at 305 d and the reproductive parameter Daughter Calving Ease, the first two being higher in cows of the HI group and the third lower in the LI group (*p* < 0.05). For the production phenotypic parameters, statistically significant differences were observed between both groups in the Total Fat, Total Protein, and Urea parameters, the first two being higher in the LI group (*p* < 0.05). Also, significant differences were observed in several reproductive phenotypic parameters, such as Number of Services per Conception, Calving to Conception Interval, Days Open Post Service, and Current Inter-Partum Period, all of which negatively influenced the HI group (*p* < 0.05). In addition, correlation analyses were performed between production and reproductive genomic parameters separately and in each consanguinity group. The results showed multiple positive and negative correlations between the production and reproductive parameters independently of the group analyzed, being these correlations more remarkable for the reproductive parameters in the LI group and the production parameters in the HI group (*p* < 0.05). In conclusion, the degree of inbreeding significantly influenced the results, affecting different genomic and phenotypic production and reproductive parameters in high-producing primiparous cows. The determination of the II in first-calf heifers is crucial to evaluate the negative effects associated with homozygosity avoiding an increase in inbreeding depression on production and reproductive traits.

## 1. Introduction

In the last decade, the application of genomic evaluation in dairy cattle has been a crucial tool for the analysis and selection of suitable individuals thus ensuring better productivity. The technology has led to important changes in countries with dairy traditions, directly affecting the benefits of the world dairy industry [1].

Before the emergence of genomics, information to predict production and reproductive patterns was based on progeny selection tests that took long generation intervals [2]. Fortunately, the current trend is to perform evaluations based on genotyping of singlenucleotide polymorphisms (SNPs) which account for nearly 75% of reliability [3]. Nowadays, the number of dairy cows genotyped for heritable traits (e.g., productive, reproductive, fitness, and conformation) is increasing [1]. This is because genomic profiles have become more reliable for predictions [4,5]. For these analyses, high-density SNP chips are used with one- or two-step frequentist and Bayesian methodologies [6]. The information generated can be used to build data banks and for the exchange of specific genotypes among countries interested in improving productive and reproductive herd traits, conforming, for instance, the inter-genomic database such as Interbull (Interbull Centre, Uppsala, Sweden) [3,7,8].

In the highly demanding dairy cattle industry, genetic improvement depended for decades on the use of pedigree and phenotype data to estimate breeding and production values [9]. However, at present, scarce knowledge is available about the genetic architecture of quantitative variation regarding features such as inbreeding and its influence on production and reproductive parameters [2,5,10]. Accordingly, the implementation of genomic evaluations can include more relevant parameters, such as inbreeding, with the potential of significantly affecting selection indices, with emphasis on milk production, milk characteristics, reproduction, conformation traits, and productive lifespan [11,12]. Health parameters, selection for organic production, reduced waste, and gas emission are also currently considered traits of interest [13,14]. Therefore, at present, it becomes necessary to add new parameters to genotype analyses to select animals with more accurate estimates of SNPs’ effects that directly or indirectly affect profitability [15]. Consequently, routine genotyping of primiparous cows and the incorporation of new indices such as inbreeding can significantly aid in making better management decisions in herds [4,16].

Genotyping is currently focusing on the selection of the female animals that should be kept in herds, increasing the information derived from estimates in young animals [7,8]. Thus, future genomic evaluation algorithms will be based on one-step methodologies single–step Genomic Best Linear Unbiased Prediction (ssGBLUP) that will incorporate hundreds of thousands of cows with genotypes and trait records [17,18] and that will gradually replace the pedigree relationship matrix with genomic relationships [2].

Inbreeding occurs when crossing genetically related animals because they share common ancestors [9]. Inbreeding in farm animals reduces genotypic variability by restricting heterozygosity [16,19] with consequently an increase of deleterious recessive genes’ frequency visible in a lower phenotypic variability [20]. Furthermore, inbreeding can lead to a reduction in genetic variability with possible harmful genetic and phenotypic effects [20]. Therefore, the study of inbreeding in dairy cattle is extremely important, particularly due to the widespread use of assisted reproductive technologies (ARTs) that produce high selection pressure and rapid genetic gain, resulting in thousands of offspring from a single parent [21].

A great effort has been made in the genotyping of dairy cattle of the Holstein breed, but in general, this task has been insufficient. A reduction of convergence issues via increasing the proportion of pre-selected animals genotyped in a population will be decisive in the detection of genotypes with no significant values of inbreeding [22]. This will solve the current problem of scarce genetic ties among sires of different bloodlines [23]. The physiological consequences of high milk yields and low reproduction rates (e.g., fertility) per cow during the last 20 years are still a strong challenge. Therefore, the determination of new polymorphic selection genes for high-yielding cattle is increasingly relevant in our times. These evaluations should rely more on nobel research involving functional parameters including inbreeding parameters together with production and reproductive parameters [24].

There are several reports about the effects of inbreeding on some production parameters, such as milk production (kg/305 d) or the percentage of fat and protein in milk [25,26,27,28,29,30,31,32]. According to some authors, it has been estimated in Holstein that, on average, per every 1% of inbreeding, the mean of milk/animal/day has a decrease of ~5 L and the mean of total milk production per lactation a decrease of ~200 kg [33]. However, this work was done when genomic evaluation techniques were not yet fully developed. Considering the evident negative effect of inbreeding on production and reproductive parameters, more in-depth research analyzing the effect of genomic inbreeding indexes on phenotypic production and reproduction indexes in high-producing primiparous dairy cows is needed. The information obtained could contribute to elucidate the underlying genomic architecture estimated through genetic correlations and variability in the traits as those mentioned above when inbreeding is present [34]. Thus, it is necessary to overcome the challenges posed by the predictive biology of parameters and indicators required to develop effective genomic selection programs in dairy cattle, considering the possible negative effects of inbreeding. Therefore, the objective of this research was to study the application of the inbreeding index (II) (low and high) based on genomic analysis and its effect on production and reproduction-related genotypic and phenotypic parameters in high-yielding primiparous dairy cows.

## 2. Materials and Methods

### 2.1. Ethical Statement

The authors declare that the present study was carried out by the following the Code of Ethics for animal experiments as reflected in the ARRIVE guidelines available at http://www.nc3rs.org.uk/ARRIVEchecklist. This study was approved by the Bioethics Committee for the use of experimental animals at the Universidad de Concepción—Campus, Chillan—Chile (Approval Date: 01/09/2018, Code Number: CBE-8269/2018).

### 2.2. Location, Environmental Conditions, and Animals

The study was carried out in the Ñuble region (Chile), South zone 36°4432″ S 72°1755″ W. This region has annual temperatures that fluctuate between 13.5 °C and 14 °C with a maximum in the hottest month (January) of 33 °C and an average minimum in the coldest month (July) between −2 °C and 5 °C. Annual rainfall is 1025 mm. The study was conducted between July 2017 and June 2020. Handling, feeding, and health conditions of the herd were standardized according to the requirements of high-producing dairy cows (NRC).

High-producing Holstein–Friesian cows (*Bos taurus taurus*, *n* = 3209) were selected. From that population, only those animals at first calving were selected for this study (*n* = 385). After an initial selection of animals based on production and udder health characteristics using Research Randomized (RR v.4.0, SPN, Pennsylvania, PA, USA) and G*Power (v.3.1.9.7, Universität Düsseldorf, Düsseldorf, Germany) for random sampling and random assignment tools; 100 animals were used, divided into 2 groups: one with low inbreeding (LI: <2.5%) and one with high inbreeding (HI: ≥2.5% and ≤5.0%) according to genomic inbreeding traits. The classification of both groups was carried out based on previous approaches including the assessment of thousands of cows and establishing an inbreeding coefficient of ~2.6 on average for Holstein breed [16,26,27]. Moreover, the highest threshold was established on 5.0 (two-fold), based on Dezetter et al. [29] observations to avoid possible outliers due to the extremely high inbreeding index values (II > 5.0). Regarding animal selection by production characteristics, having into account that a Holstein primiparous cow is on average ~24–26 L/d [35,36], only high-producing individuals above this threshold (+20% L/d) were selected. Finally, after leaving out some animals with unspecific problems, a total of 80 high-producing cows were used based on the number of births (primiparous) with an average age between 23 and 26 months with ≥30 L average milk/cow/day, adjusted to 305 days of lactation. Genome trait data were obtained from Zoetis–Clarifide, (San Diego, CA, USA) which provides genomic evaluations for dairy cattle. Data on phenotypic traits were obtained from first lactation production records and breeding records associated to first calving in each genotyped animal and recorded in the farm’s milk control software (DairyComp 305, Winnipeg, MB, Canada). The yields of milk, fat, protein, and somatic cells were standardized to 305 lactation days. This study included records of all nulliparous cows that became pregnant and calved regardless of whether conception occurred at first or more inseminations.

### 2.3. Genomic Analysis

**Figure 1:**
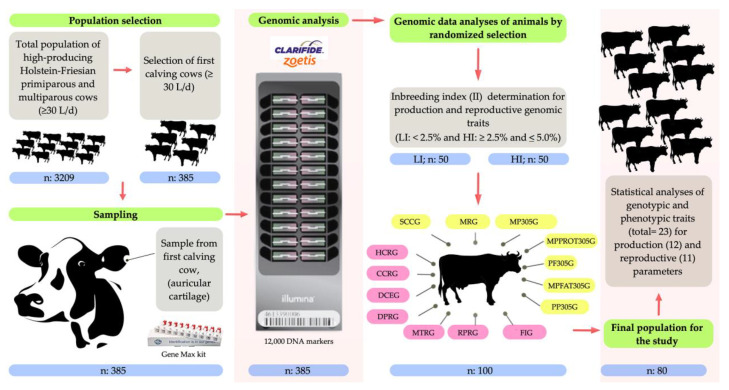
Experimental design map. Dairy cows used for the Inbreeding Index (II) determination (ranges <2.5% and ≥2.5% to ≤5.0) from the total population analyzed (*n* = 385) by genomic tests (genotypic and phenotypic parameters). For genomic analysis, the sampling was carried out by using the Gene Max kit (TSU, ALLFLEX, Kalamazoo, MI, USA) and next a chip with 12,000 DNA markers (12K; Illumina Bovine SNP50 BeadChip, Clarifide–Zoetis, San Diego, CA, USA) was used for genotyping. The results have a ~70% reliability based on a panel of 50,000 (HD50K) genetic markers from the Animal Improvement Programs Laboratory (AIPL) (Zoetis–Clarifide, SanDiego, CA, USA). Genomic production values: MP305G: Milk Production at 305 days; MPFAT305G: Fat Production at 305 days; MPPROT305G: Protein Production at 305 days; PF305G: Percent of Fat at 305 days; PP305G: Percent of Protein at 305 days; SCCG: Somatic Cell Count; MRG: Mastitis Resistance. Genomic reproductive values: FIG: Fertility Index; HCRG: Nulliparous Cow Conception Rate; DPRG: Daughter Conception Rate; CCRG: Cow Conception Rate; DCEG: Daughter Calving Ease; MTRG: Resistance to Metritis; RPRG: Resistance to Placental Retention.

For the genomic analysis, the selected cows were studied with genomic tests (Illumina Bovine SNP50 BeadChip, Clarifide–Zoetis, San Diego, CA, USA). For the genotyping sampling, the Gene Max kit (TSU, ALLFLEX, Kalamazoo, MI, USA) was used to take samples from each nulliparous cow (heifer) ear (pinna). A chip with 12,000 DNA markers (12K) was used, which provides the most relevant information regarding production, reproduction, health, and type-related characteristics with a ~70% reliability based on a panel of 50,000 (HD50K) genetic markers from the Animal Improvement Programs Laboratory (AIPL) (Zoetis–Clarifide, San Diego, CA, USA). The information obtained predicts animal behavior based on the most economically relevant dairy traits and have been widely used for the characterization of elite sires and sire mothers in the dairy industry (Clarifide™ Dairy, Zoetis Genetics, San Diego, CA, USA). The initial genomic analysis (screening) allowed to identify and characterize those animals above the average Inbreeding Indices (II) and those below (between 1–10 points and ranges <2.5% and ≥2.5% to ≤5.0) to later correlate genotypic and phenotypic productive-reproductive traits (Figure 1).

### 2.4. Analysis of the Inbreeding Index (II)

Both genotypic and phenotypic parameters (production and reproductive) obtained were compared against the genomic individual Inbreeding Index (II). The II values estimate the current homozygosis and the percentage (%) of gene regions in common instead of individual expected inbreeding fractions calculated from the genealogy (pedigrees). The reference population represents all genotyped animals born in the last 10 years, with values close to zero the most desirables.

### 2.5. Analysis of Genomic Parameters for Production Traits

The genomic parameters for production traits that were evaluated are shown in detail in Table S1. They were: Milk Production (MP305G; lbs/lactation): describes genetic differences in total pounds of milk produced at 305-day lactation; Protein Percentage (PP305G; %) describes genetic differences in % of milk protein; Fat Percentage (PF305G; %) describes genetic differences in % of milk fat; and Somatic Cell Count (SCCG; log2-Score) indirectly predicts susceptibility to mastitis. The somatic cell count was transformed (SCS = (log2 (RCS/100,000) + 3) to improve the normality of the data. Other parameters evaluated were total protein (MPROT305G; lbs) which evaluates genetic differences in the amount of protein produced during a 305 day lactation; total fat (MPFAT305G; lbs) which evaluates genetic differences in the amount of fat in milk produced at 305 day lactation period and mastitis resistance (MRS; %) which measures an animal’s genetic resistance to mastitis. High values are consistent with animals with greater resistance to mastitis.

### 2.6. Analysis of Genomic Parameters for Reproductive Traits

The genomic parameters for reproductive traits that were evaluated are shown in detail in Table S2. They were: Fertility Index (FIG), that measures the reproductive efficiency in a female or a group of animals incorporating several specific characteristics (such as the following parameters): Heifer’s Conception Rate (HCRG; %) which measures the increase of the percentage for the nulliparous cow (heifer) conception, defined as the % of nulliparous cows (heifers) inseminated becoming pregnant in each service; Daughter Pregnancy Rate (DPRG; %) is a measurement of reproductive efficiency and estimates the percentage of cows eligible to become pregnant in a 21 day period (average duration of the estrous cycle) that become pregnant (expected percentage difference with respect to the breed average); Cow Conception Rate (CCRG; %) measures possible gains in the pregnancy percentage of lactating cows (% of inseminated cows that become pregnant on each service). Daughter Calving Ease (DCEG; %) measures percentual genetic gains for cows that calve easily (% of non-dystocia in first calving cows); Metritis Resistance (MTRG; %) measures the genetic percentage of resistance to metritis; Resistance to Placental Retention (RPRG; %) measures the genetic percentage of resistance to placental retention.

### 2.7. Analysis of Phenotypic Parameters for Production Traits

The phenotypic parameters for production traits that were evaluated are shown in detail in Table S1. Data were collected on the phenotypic production traits of the milk control program adjusted to 305 days of lactation. Parameters were: Total Liters of Milk (TLM; l) which is the total liters of milk produced during a 305 day lactation; Total Fat (TFAT; lb) measures the amount of fat produced during a 305 day lactation; Total Protein (TPROT; lb) measures the amount of protein produced during a 305 day lactation; Milk Projected at 305 days (MPJ305; l) estimates the amount of milk projected for production during a 305 day lactation; Average Liters of Milk (ALM; l) is the average milk produced per day; Fat Percent (FATP; %) is the percentage of fat in produced milk; Protein Percent (PROTP; %) is the percentage of protein in produced milk; Liters of Current Corrected Milk (LCCM; l) is the amount of current milk produced corrected to a lactation; Milk Projection 305 days Maturity Equivalent (MPJ305ME; l) estimates the projected milk production at 305 days which is equivalent to accumulated production maturity in each month; Somatic Cell Count (SCC; cells × 10^3^/mL) is the value of somatic cell numbers per mL throughout the lactation; Logarithm of Somatic Cell Count (LGSCC; log2) is the value of the adjusted somatic cell number in a lactation; UREA (U; mg/mL) is the value that reflects the urea nitrogen in relation to a correct energy/protein balance. Milk samples (15 mL) were taken daily during the morning milking (6:00 a.m.). Protein percentage, fat percentage, and somatic cell count were analyzed by specialized testing equipment (CombiFoss TM 7 3400, Fossomatic, Denmark) based on Flow Cytometry, using specific fluorescent probes that provide real-time chemical and structural information of the milk components according to their different luminescent distribution patterns with corrective standardization parameters (calibration ranges up to 50% in fat and 7% in protein, the accuracy of ≤0.8% CV) on main components of raw cow’s milk (i.e., fat, protein) and with a homogeneous sample analysis temperature (22–23 °C).

### 2.8. Analysis of Phenotypic Parameters for Reproductive Traits

Details of the phenotypic parameters for reproductive traits that were evaluated are shown in Table S2. They were those specific for estimating fertility and reproductive efficiency such as Weight at First Service (WFS; kg) that measures the optimal heifer weight at first service, considering the cattle breed. Another relevant reproductive variable is Number of Services per Conception which can be evaluated through several parameters as follows. Heifer (NSCNC; points) measures the average number of precise inseminations in cows; Number of Services per Conception in cows (NSCC, points) measures the average number of precise inseminations in cows to obtain a pregnancy. Other reproductive parameters were: Days of Gestation (DG; days) measures the duration of pregnancy in cows; Days in Milk at First Breeding (DMFB; points) is the number of days in lactation from calving to the first service; Days in Anestrus Post Voluntary Waiting Period (DAPVWP; days) measures the number of post-partum days from voluntary waiting time to the first insemination; Calving to Conception Interval (CCI; days) is the period from calving to effective pregnancy; Days Open Post Service (DOPS; days) period between the first insemination and the fertilizing insemination; Efficiency in Heat Detection (EHD; %) is the percentage with which heat is detected in a period of 21 days obtained by dividing 21 by the average interval between heats × 100 ((21/IPEC) × 100); Minimum Projected Inter-Partum Period (MPJIPP; days) is the adjusted projected time between one delivery to the next; Current Inter-Partum Period (CIPP; days) is the period between one delivery to the next (Table S2).

### 2.9. Statistical Analysis

The statistical analysis of the data was carried out using SPSS v.20 software for Windows. The mean and standard error of the mean were calculated for descriptive statistics. After a previous exploration of the data matrix employing the Kolmogorov–Smirnov test and Q–Q plots tests to determine the normality of residuals distributions, one-way ANOVA test was performed to compare the means obtained from the genomic (i.e., genotypic and phenotypic) production and reproductive parameters. When statistically significant differences were detected, analyses based on parametric correlations using Pearson’s linear correlation tests were carried out to study the relationship between pairs of production and reproductive genomic parameters. Moreover, a test based on parametric linear correlations was carried out to study the relationship between pairs within genotypic production parameters and between pairs within genotypic reproductive parameters. Finally, following the same procedure, a test based on parametric linear correlations was carried out to study the relationship between pairs within phenotypic production parameters and between pairs within phenotypic reproductive parameters. The differences were considered statistically significant when *p* ≤ 0.05.

## 3. Results

### 3.1. Analysis of Genomic Parameters for Production Traits

**Figure 2:**
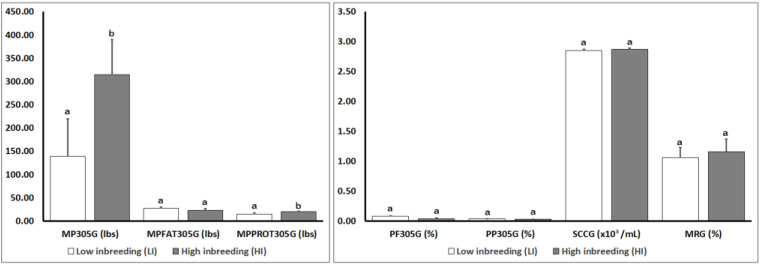
Effect of the degree of inbreeding (LI versus HI) on the genomic parameters for production traits in high-producing primiparous dairy cows. Production genomic values: MP305G: Milk Production at 305 days; MPFAT305G: Fat Production at 305 days; MPPROT305G: Protein Production at 305 days; PF305G: Percent of Fat at 305 days; PP305G: Percent of Protein at 305 days; SCCG: Somatic Cell Count; MRG: Mastitis Resistance. Different letters show statistical differences between values (*p* ≤ 0.05). lbs: Pounds; %: Percentage; SEM: Standard Error of the Mean.

Figure 2 shows detailed results of the genomic parameters for production traits in both inbreeding groups (LI and HI) in the present study. Genomic parameters for production traits were similar between the LI and HI groups with no statistically significant differences in any production genomic parameter (*p* > 0.05), except for the parameters MP305G (*p* = 0.05) and MPPROT305G (*p* = 0.02) in which statistically significant differences were observed between the LI and HI groups.

### 3.2. Analysis of Phenotypic Parameters for Production Traits

**Figure 3:**
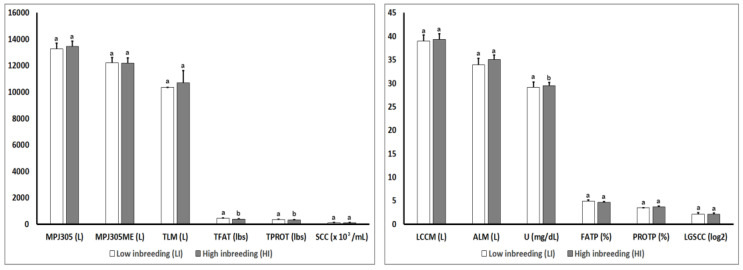
Effect of the degree of inbreeding (LI versus HI) on phenotypic parameters for production traits in high-producing primiparous dairy cows. Production phenotypic values: MPJ305: Projected Milk at 305 days; MPJ305ME: Projected Milk 305 days Maturity Equivalent; TLM: Total Liters of Milk; TFAT: Total Fat; TPROT: Total Protein; SCC: Somatic Cell Count; LCCM: Liters of Current Corrected Milk; ALM: Average Liters of Milk; U: Urea; FATP: Percentage of Fat; PROTP: Percentage of Protein; LGSCC: Logarithmic Somatic Cell Count. Different letters show statistical differences between values (*p* ≤ 0.05). L: Liters; lbs: Pounds; %: Percentage; mg: milligrams; dL: deciliters; SEM: Standard Error of the Mean.

Figure 3 shows detailed results of the phenotypic parameters for production traits in groups (LI and HI) in the present study. Regarding phenotypic parameters for production traits (Figure 3), statistically significant differences were observed in TFAT (*p* = 0.02), TPROT (*p* = 0.05), and U (*p* = 0.013) parameters when both groups of animals with different levels of inbreeding (LI and HI) were compared.

### 3.3. Analysis of Genomic Parameters for Reproductive Traits

**Figure 4:**
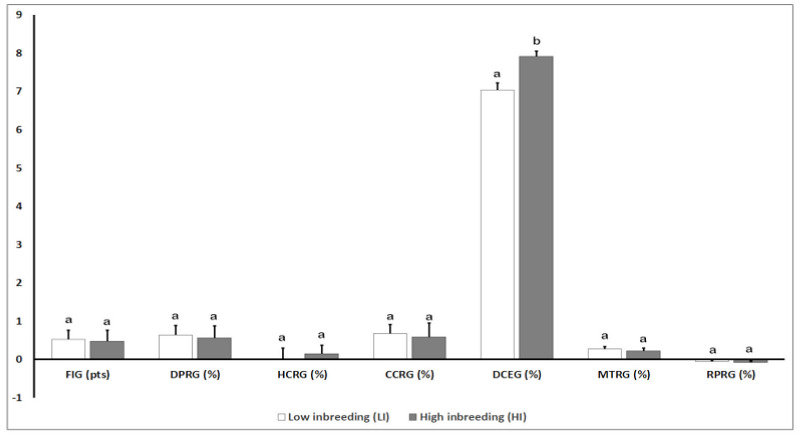
Effect of the degree of inbreeding (LI versus HI) on genomic parameters for reproductive traits in high-producing primiparous dairy cows. Reproductive genomic values: FIG: Fertility Index; DPRG: Daughter Conception Rate; HCRG: Nulliparous Cow Conception Rate; CCRG: Cow Conception Rate; DCEG: Daughter Calving Ease; MTRG: Resistance to Metritis; RPRG: Resistance to Placental Retention; NS: Not Significant; SEM = Standard Error of the Mean. Different letters show statistical differences between values (*p* ≤ 0.05). pts: points; %: Percentage; SEM: Standard Error of the Mean.

Figure 4 shows detailed results of the genomic parameters for reproductive traits in the study groups (i.e., LI and HI). Concerning genomic parameters for reproductive traits, statistically significant differences were only observed in the DCEG parameter when both groups (i.e., LI and HI) were compared (*p* = 0.05).

### 3.4. Analysis of Phenotypic Parameters for Reproductive Traits

**Figure 5:**
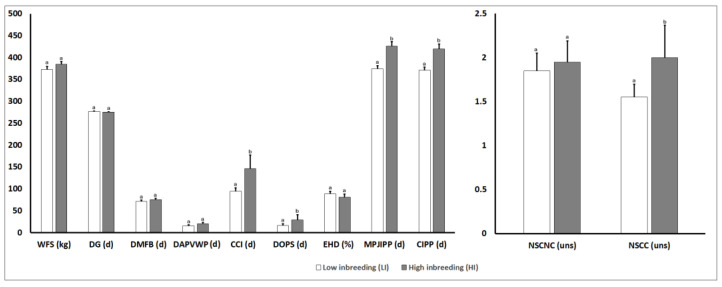
Effect of the degree of inbreeding (LI and HI) on phenotypic parameters for reproductive traits in high producing primiparous dairy cows. Reproductive phenotypic values: WFS: Weight at First Service; DG: Days of Gestation; DMFB: Days in Milk at First Service; DAPVWP: Days of Anestrus Post Voluntary Waiting Time; CCI: Calving to Conception Interval; DOPS: Days Open Post Service; EHD: Efficiency in Heat Detection; MPJIPP: Minimum Projected Inter-Partum Interval; CIPP: Current Inter-Partum Period. NSCNC: Number of Services per Conception: Nulliparous Cow (heifer); NSCC: Number of Services per Conception in Cows Different letters show statistical differences between values (*p* ≤ 0.05). Kg: Kilograms; uns: Units; d: days; %: Percentage; SEM: Standard Error of the Mean.

Figure 5 shows detailed results of the phenotypic parameters for reproductive traits in both inbreeding groups (LI and HI) obtained in the present study. Regarding phenotypic parameters for reproductive traits when both inbreeding groups (LI and HI) were compared, statistically significant differences were observed in NSCC (*p* = 0.03), CCI (*p* = 0.001), DOPS (*p* = 0.05), MPJIPP (*p* = 0.001), and CIPP (*p* < 0.001).

### 3.5. Linear Correlation Analyses between Genomic Parameters of Production and Reproductive Traits

Multiple statistically significant correlations between parameters within the LI and HI groups were observed regarding genomically analyzed production–reproductive traits (*p* < 0.05; Table 1 and Table 2). Specifically, moderate negative correlations were observed between parameter pairs MP305G–FIG, MP305G–DPRG, MPPROT305G–FIG, and MPPROT305G–DPRG in the LI group (*p* < 0.05; *p* ≤ 0.01; Table 1). Regarding positive correlations, no statistically significant correlations were observed between the production and reproductive genomic parameters in the LI group (*p* > 0.05; Table 1). Moreover, stronger significant negative correlations were observed between parameter pairs MP305G–FIG, MP305G–DPRG, MP305G–HCRG, MP305G–CCRG, MPPROT305G–FIG, MPPROT305G–DPRG MPPROT305G–CCRG, and SCCG–RPRG in the HI group (*p* < 0.05; *p* ≤ 0.01; Table 2). Also, significant positive correlations were observed between productive and reproductive genomic parameters PF305G–DPRG, PF305G–HCRG, MRG–FIG, MRG–PDRG, MRG–CCRG in HI group (*p* < 0.05; *p* ≤ 0.01; Table 2).

Table 2 shows detailed results of the correlation analysis between production and reproductive genotypic parameters obtained within the HI group. An increase in the number of positive and negative correlations within HI group was observed, as well as an increase in the correlation significance between production/reproductive parameters when the degree of inbreeding increased.

No statistically significant positive or negative correlations were observed regarding the rest of the non-mentioned production and reproductive parameters analyzed, independently of the group (LI or HI) of animals evaluated (*p* > 0.05) indicating that the effects of the level of inbreeding do not have an important influence on the correlation between these parameters, independently of the group analyzed (Table 1 and Table 2). As observed, individuals with II ≥ 2.5 and ≤5.0 showed up to a two-fold increase in the negative correlations between genomic production and reproductive parameters when HI and LI were compared (Table 1 and Table 2) affecting seriously important traits such as Milk Production at 305 d (MP305G) and Protein Production at 305 d (MPPROT305G) versus Fertility Index (FIG) and Daughter Pregnancy Rate (DPRG), respectively (*p* < 0.05).

### 3.6. Linear Correlation Analyses within Genomic Parameters of Production and Reproductive Traits

In LI group high/moderate and statistically significant positive correlations within production (MP305G–MPPROT305G, *r*: 0.817, *p* = 0.0001; MPFAT305G–PF305G, *r*: 0.512, *p* = 0.021; PF305G–PP305G, *r*: 0.538, *p* = 0.014) and reproductive (FIG–DPRG, *r*: 0.971, *p* = 0.0001; FIG–HCRG, *r*: 0.755, *p* = 0.0001; FIG–CCRG, *r*: 0.909, *p* = 0.0001; DPRG–HCRG, *r*: 0.593, *p* = 0.006; DPRG–CCRG, *r*: 0.849, *p* = 0.0001; HCRG–CCRG, *r*: 0.654, *p* = 0.002) parameter pairs were observed, being the most outstanding positive correlations in the LI group. Moreover, high/moderate and statistically significant negative correlations were observed within production (MP305G–PF305G, *r*: −0.747, *p* = 0.0001; MP305G–PP305G, *r*: −0.550, *p* = 0.012; MPPROT305G–PF305G, *r*: −0.509, *p* = 0.022; MRG–SCCG, *r*: −0.580, *p* = 0.007) and reproductive (FIG–DCEG, *r*: −0.484, *p* = 0.031; DCEG–HCRG, *r*: −0.530, *p* = 0.016; HCRG–RPRG, *r*: −0.572, *p* = 0.008) parameter pairs, being the most outstanding negative correlations in the LI group.

In addition, high/moderate positive correlations were observed between production (MP305G–MPPROT305G, *r*: 0.690, *p* = 0.001; MPFAT305G–PF305G, *r*: 0.653, *p* = 0.002; PF305G–PP305G, *r*: 0.589, *p* = 0.006) and reproductive (FIG–DPRG, *r*: 0.986, *p* = 0.0001; FIG–CCRG, *r*: 0.969, *p* = 0.0001; FIG–HCRG, *r*: 0.541, *p* = 0.014; DPRG–CCRG, *r*: 0.936, *p* = 0.0001; CCRG–HCRG, *r*: 0.460, *p* = 0.041) parameter pairs in the HI group and high/moderate negative and statistically significant correlations were found in the MP305G–PF305G (*r*: −0.659; *p* = 0.002), MP305G–PP305G (*r*: −0.846; *p* = 0.0001) and MRG–MPFAT305G (*r*: −0.526; *p* = 0.017) parameter pairs, being these negative correlations the most outstanding in the HI group.

## 4. Discussion

The aim of the present research work was to evaluate the impact of the inbreeding degree in parameters related to production and reproductive parameters in high-producing primiparous dairy cows. Several research groups have found differences in the effect of the degree of inbreeding on the parameters studied, showing adverse effects on different phenotypic and genotypic traits in dairy cattle, mainly due to the reduction of genetic variability [10,27,29,31,37,38,39]. In contrast to other authors who in the mid-1980s claimed that the impact of reduced genetic variability on dairy cattle was negligible; more recent work indicates that reduced genetic variability may have a highly negative impact on livestock productivity [40,41]. In our study, we observed that the effect of inbreeding has a differentiated impact depending on the specific phenotypic/genotypic parameter studied. The fact that phenotypic parameters are differentially affected indicates the need to go further in the evaluation of the animals using genomic tools to determine the impact of inbreeding depression (ID) thus allowing a more accurate estimation of such impact [39].

It has been estimated that, on average, per every 1% of inbreeding, the mean of a production/reproductive parameter has a decrease of 0.137% [42]. However, there are no studies to find a correct threshold to define a significant value for production and reproductive parameters in high-producing primiparous cows. Therefore, the present research compared animals with different levels of inbreeding (LI: <2.5% and HI: ≥2.5 and ≤5.0) to determine the impact on genotypic/phenotypic, production/reproductive parameters in primiparous cows with lactations adjusted to 305 days.

Regarding genomic parameters linked to production, the only observed parameters in this study showing significant differences when comparing inbreeding groups (i.e., LI and HI) were MP305G and MPPROT305G (milk production at 305 days and protein production at 305 days, respectively). In particular, we found a negative effect on milk and protein production adjusted to 305 days when comparing the LI with HI groups of high-producing primiparous cows. This is in agreement with Dezetter et al. [29] and Howard et al. [9] who showed that inbreeding harmed production performance in different breeds and crosses, particularly in multiparous Holstein cows at 305 days of lactation [39]. Furthermore, it has also been observed that a 1% increase in the inbreeding index is associated with a decrease in milk production yields of around 0.6% on average [28]. Consequently, according to our study and in accordance with previous work, an increase in the levels of genomic inbreeding would be associated with negative effects on milk production yield at 305 days.

Similarly to our findings on production phenotypic parameters TFAT and TPROT (significantly affected by the degree of inbreeding), several research groups have found that the degree of inbreeding in purebred multiparous Holstein–Friesian cows and crosses negatively affects production traits, such as a decrease of 12.5% of milk production obtained by lactation and adjusted to 305 days, and in fat and protein percentages, accompanied by an increase in the SCC-log [16,26,27]. These results were corroborated in the present study, since we observed that in purebred Holstein–Friesian primiparous cows, although a decrease in milk production at 305 d (total Kg/lactation) was associated with a higher degree of inbreeding, it was neither significantly different between groups nor was the SCC-log. These observations contrast with the work of Doekes et al. [41] for milk production and Martikainen et al. [31] for milk production and SCC%. However, the latter study was conducted on Ayrshire dairy cattle which may be differentially affected due to the breed factor. Thus, in general, these studies agree that there is a decrease in productive performance due to the effect of inbreeding on milk production traits such as Total Liters of Milk (TLM), TFAT, and TPROT, similar to the results obtained in our study except for TLM and increased values of SCC. In our study, we found neither a decrease in TLM nor an increase in SCC possibly because these parameters could be negatively affected in subsequent lactations due to the stabilization of productivity and increases in the inbreeding coefficient [27]. Another explanation is the fact that some breed traits, crosses, genetic lines, or isolated animal populations with different degrees of inbreeding could be differentially more affected by ID than others [42].

Another parameter affected by the degree of inbreeding in the present work was blood urea concentration. A dietary imbalance between energy/raw protein produces reproductive and production problems [43]. In general, the variability of blood urea levels is high and depends on intrinsic (genetic) and extrinsic (dietary) factors [44,45]. Results published by other authors showed that as milk production increases, urea levels increase and SCC levels decrease, suggesting that selection based on production performance increases the urea levels and therefore it can be considered a characteristic feature of high-producing animals [46]. However, in the present study, in spite of the urea increasing significantly in HI compared to LI, the increase of TLM was not significantly different between groups.

The only genomic-associated, reproductive parameter affected by the degree of consanguinity was DCEG. This parameter is important because it is part of the total Fertility Performance Index (FIG). It shows the tendency to have more or fewer problems during delivery in the female offspring of a given sire. In the present study, differences were observed between the LI and HI groups, being DCEG in the latter group a strongly affected parameter. This fact was corroborated by other authors who determined that inbreeding rates have a negative influence on the ease of delivery [27,37]. In contrast, other studies found that the degree of inbreeding did not have a significant effect on ease of calving, although they did show a significant effect on fetal death [47,48,49]. Even though these studies seem to be contradictory, we speculate that the risk of fetal death may affect the prevalence of difficult deliveries. One possible cause would be the absence of fetal signals for labor initiation resulting in uterine atony and subsequent dystocia [50].

Regarding the effect of inbreeding on reproductive phenotypic parameters, several studies have observed a deterioration in fertility as a gradually increasing problem over the last few decades, especially in dairy cattle [10,16,26,27,31,37,47,48,51,52,53]. In the present work, carried out in high-producing primiparous cows, the effect of the degree of inbreeding was reflected in the NSCC, a fact that shows a greater deterioration of fertility in animals belonging to the HI group. Studies in purebred Holstein–Friesian cows and crosses have shown a decrease in fertility traceable to the need for more inseminations per animal (up to 1 more per multiparous cow) [29]. In the present work, this fact is particularly evident in primiparous cows, indicating a notable effect of the degree of consanguinity on fertility, independently of the number of births. According to Martikainen et al. [31], homozygosity derived from heritability of identical haplotypes from both parents is associated with ID, adversely affecting fertility traits. Another parameter affected in the present study was the CCI, which is influenced by environment, husbandry and genotypic factors [26]. Considering that in our study all the animals were handled similarly and kept in the same environment, we hypothesize that the deterioration of the CCI may be due to a higher degree of inbreeding as reflected by the animals of the HI group. In studies conducted on other breeds, such as Ayrshire, increases of up to 17 days in the CCI were observed [51]. In the present study involving primiparous Holstein cows, high inbreeding (HI) levels generated significant differences for the CCI trait (days), although not to the same extent as in the work of Martikainen et al. [31], which could be due to the breed differences. Nevertheless, this effect could be further expressed in subsequent lactations which would increase the number of days in the CCI, reducing the productive life of the animal and causing significant economic losses.

Another reproductive parameter affected in the present study was DOPS. Likewise, several studies have described a link between the degree of consanguinity and a decrease in reproductive capacity due to the increase in the interval between inseminations [37]. Consanguinity directly affects the viability of the embryo which could generate early embryonic losses, thus affecting DOPS [51]. Other authors have reported haplotype lengths in genomic regions harboring harmful recessive mutations in animals of the Holstein, Montbéliarde, and Normande breeds with a significant negative effect on birth rate [48]. Together, all these factors that increase DOPS in post-service multiparous dairy cows may be caused by the effect of ID associated with different levels of inbreeding, as reflected in the present work on high-producing primiparous cows.

Efficiency in Heat Detection (EHD) is an important indicator strongly associated with fertility in dairy cattle herds. The EHD value should be close to 70% of animals observed in estrus at 55 days postpartum and close to 95% at 63 days postpartum. Several authors indicate that variability in EHD may be due to the fact of human error or pathological anestrus due to the negative genomic effects [54,55,56,57,58,59,60]. In the present work, no significant difference was observed in EHD, although this is a very important feature to consider within the fertility index, since poor or no oestrus expression has been suggested to be linked to lower fertility. This fact is in agreement with Martikainen et al. [31] who indicated that Runs of Homozygosity (ROH) favor the harmful recessive alleles generated by inbreeding that affect fertility. Therefore, we could consider that as lactations develop, the greater cumulative effect of the inbreeding index and, therefore, a greater ID will induce lower fertility, a softer manifestation of oestrus and lower EHD.

In the present work, we realized that different levels of inbreeding (LI and HI) generate different effects on several reproductive parameters. Variation in CIPP (CIPP measures the elapsed time from one birth to the next) is associated with the degree of inbreeding. However, an increase in inter-calving days could be strongly influenced by reproductive performance as well as husbandry. Therefore, according to the results of this study, the genetic factor is associated with inbreeding and CIPP increases as the environment and management of the animals are suboptimal. Several environmental and husbandry factors may be associated with alterations in CIPP. However, the degree of inbreeding may add to these factors. Different authors have estimated an increase in the interval between births due to the undesirable effects from inbreeding [26,30]. In contrast, Rokouei et al. [27] observed that the undesirable effects of inbreeding were not significant for other reproductive traits, but CIPP in particular increased by 1% from the third birth on and so did the age at first calving [27]. Effects, such as breed (gestation length) and differences in the number of births (primiparous versus multiparous), could also influence CIPP.

Linear correlation analyses of genomic parameters connecting production and reproductive traits of the LI and HI groups showed significant positive (LI) as well as negative (HI) trends, respectively. In these two groups, it was observed a predominant influence of economically productive traits such as MP305G and FIG over several reproductive and production traits, which are strongly associated with high-producing dairy cows. Briefly, when milk production increases the concentration of total solids during the lactation increases as well, and vice versa. Likewise, we observe that when the FIG index increases, the associated traits also increase and vice versa. This is consistent with different studies that evaluated the effects of recent and old inbreeding on production performance, fertility, and health traits in Holstein cows [30,39]. These authors concluded that inbreeding decreases animal performance, but depending on the parameters evaluated, the inbreeding effects might be equally detrimental for other parameters.

In addition, our study shows negative correlations in both groups studied (LI and HI) concerning parameters linked to production and FIG (MP305G–FIG and MPPROT305G–FIG), despite the increase in the variability of the parameters as the degree of consanguinity increased. This may be due to an effect of the high productions reached by Holstein cows and the breed factor linked to inbreeding, a fact that has been reported as high inbreeding coefficients in dairy breeds [61,62]. Moreover, these negative correlations may have also been caused by additive and dominant genetic variations of loci for fertility traits that occur in Holstein cows [63]. Therefore, the inbred effect at different levels could be responsible for this variability in genomic traits, with negative correlations increasing in subsequent lactations, as observed in the HI group.

Negative correlations of production and reproductive traits were observed in both LI and HI groups (MP305G–DPRG and MPPROT305G–DPRG). Moreover, negative correlations of production–reproductive related traits in the HI group (MP305G–HCRG, MP305G–CCRG; MPPROT305G–CCRG, PF305G–DPRG, and PF305G–HCRG) were observed, together with health-related traits (SCCG–RPRG) which are probably linked to recessive health loci and local immunity of the uterus and the mammary gland. On the other hand, other genomic production parameters related to udder health, such as MRG, were positively correlated to genomic reproductive traits (MRG–FIG, MRG–DPRG, and MRG–CCRG), a fact in agreement with higher fertility rates and lower mastitis incidence rates. However, several studies have reported that production and fertility parameters are genetically linked, presenting negative correlations [64]. For example, as the genetic selection in dairy cows is oriented to higher yields, fertility rates are depressed [9,61]. In our study, we observed that correlations of fertility traits are associated with current production parameters in the HI group as compared to the LI group. This effect can be interpreted in the sense that as inbreeding levels increase current productive and reproduction traits may be compromised.

Finally, in the LI group, the inbreeding is low and does not generate major negative effects on production-related and reproductive-related health traits, unlike in the HI group in which greater variability in correlated parameters was observed. For example, it has been described that the magnitude and duration of the energy deficit that occurs after calving may be related to the challenge of early lactation, metabolic component, and health [65]. Therefore, the positive and negative correlations found in several production, reproductive, and health parameters in the present work would be strongly and differentially associated with the levels of inbreeding (high or low), respectively, presenting greater variability and differences of the parameters compared to the correlations in the HI group.

Regarding the results on linear correlations within production and within reproductive genomic parameters, both positive and negative correlations observed in LI and HI groups agree with the results reported in other similar studies based on the impact of the degree of inbreeding on production (milk yield, protein, and fat percentages) and reproductive (fertility index and conception rates) characteristics in multiparous dairy cows [26,29,31]. In the near future, it will be important to study the causes of variability of parameters correlated in comparisons of animals showing low and high inbreeding, including studies linking the degree of inbreeding with genotypic, phenotypic, production, reproductive, and health parameters associated to different environments. Furthermore, it would be important to identify more regions of the genome affected by inbreeding since greater or lesser differences in the phenotypic expression of the parameters mentioned could also reflect differences in the management of the animals and the strategies of the herds, which would be strongly associated with the genome and the degree of inbreeding [66].

## 5. Conclusions

High-producing primiparous dairy cows face an increased risk of negative II-derived effects in their selection programs, particularly at II ≥ 2.5. Therefore, the degree of inbreeding had an important influence affecting different genomic (phenotypic and genotypic) production and reproductive-related parameters. Characterizing the degrees of low and high inbreeding in first calving cows represents an important strategy to control the negative effects associated with homozygosity and thus to increase the ability to manage the adequate use of sires in genetic improvement programs aimed at avoiding the development of ID effects on productive and reproductive performance in subsequent lactations.

## Figures and Tables

**Table 1:** Pearson’s Correlation Coefficients between genomic parameters of production and reproductive traits obtained from primiparous high-producing dairy cows for the LI group.

Genotypic Parameters	FIG (pts)		DPRG (%)		HCRG (%)		CCRG (%)		DCEG (%)		MTRG (%)		RPRG (%)	
	*r*	*p*	*r*	*p*	*r*	*p*	*r*	*p*	*r*	*p*	*r*	*p*	*r*	*p*
**MP305G (lbs)**	−0.476	0.034	−0.565	0.010	−0.217	NS	−0.250	NS	0.027	NS	−0.128	NS	0.025	NS
**MPFAT305G (lbs)**	−0.223	NS	−0.237	NS	0.017	NS	−0.405	NS	−0.222	NS	0.338	NS	−0.123	NS
**MPPROT305G (lbs)**	−0.517	0.020	−0.593	0.006	−0.242	NS	−0.355	NS	0.168	NS	−0.002	NS	0.065	NS
**PF305G (%)**	0.260	NS	0.328	NS	0.199	NS	−0.063	NS	−0.170	NS	0.356	NS	−0.089	NS
**PP305G (%)**	0.078	NS	0.130	NS	−0.001	NS	−0.078	NS	0.210	NS	0.202	NS	0.054	NS
**SCCG (scr)**	−0.429	NS	−0.418	NS	−0.316	NS	−0.359	NS	0.119	NS	−0.169	NS	−0.015	NS
**MRG (%)**	0.205	NS	0.217	NS	0.108	NS	0.169	NS	0.198	NS	−0.038	NS	0.029	NS

Linear correlations of production genomic values: MP305G: Milk Production at 305 days; MPFAT305G: Fat Production at 305 days; MPPROT305G: Protein Production at 305 days; PF305G: Percent of Fat at 305 days; PP305G: Percent of Protein at 305 days; SCCG: Somatic Cell Count; MRG: Mastitis Resistance. Linear correlations of reproductive genomic values: FIG: Fertility Index; HCRG: Nulliparous Cow Conception Rate; DPRG: Daughter Conception Rate; CCRG: Cow Conception Rate; DCEG: Daughter Calving Ease; MTRG: Resistance to Metritis; RPRG: Resistance to Placental Retention. pts: points; lbs: Pounds; %: Percentage; scr: score; NS: Not Significant; *r*: Pearson’s correlation coefficient; *p*: Probability.

**Table 2:** Pearson’s correlation coefficients between genomic parameters of production and reproductive traits obtained from primiparous high-producing dairy cows for the HI group.

Genotypic Parameters	FIG (pts)		DPRG (%)		HCRG (%)		CCRG (%)		DCEG (%)		MTRG (%)		RPRG (%)	
	*r*	*p*	*r*	*p*	*r*	*p*	*r*	*p*	*r*	*p*	*r*	*p*	*r*	*p*
**MP305G (lbs)**	−0.556	0.011	−0.535	0.015	−0.472	0.036	−0.461	0.041	0.168	NS	−0.280	NS	−0.199	NS
**MPFAT305G (lbs)**	−0.386	NS	−0.403	NS	−0.251	NS	−0.274	NS	−0.408	NS	0.040	NS	0.289	NS
**MPPROT305G (lbs)**	−0.512	0.021	−0.488	0.029	−0.420	NS	−0.460	0.041	0.099	NS	−0.285	NS	−0.289	NS
**PF305G (%)**	0.130	NS	0.100	0.029	0.160	0.029	0.150	NS	−0.427	NS	0.248	NS	0.380	NS
**PP305G (%)**	0.360	NS	0.348	NS	0.309	NS	0.272	NS	−0.158	NS	0.193	NS	0.055	NS
**SCCG (scr)**	−0.211	NS	−0.203	NS	−0.076	NS	−0.300	NS	0.192	NS	0.188	NS	−0.569	0.009
**MRG (%)**	0.555	0.011	0.546	0.013	0.437	NS	0.488	0.029	0.208	NS	0.164	NS	0.184	NS

Linear correlations of production genomic values: MP305G: Milk Production at 305 days; MPFAT305G: Fat Production at 305 days; MPPROT305G: Protein Production at 305 days; PF305G: Percent of Fat at 305 days; PP305G: Percent of Protein at 305 days; SCCG: Somatic Cell Count; MRG: Mastitis Resistance. Linear correlations of reproductive genomic values; FIG: Fertility Index; HCRG: Nulliparous Cow (heifer) Conception Rate; DPRG: Daughter Conception Rate; CCRG: Cow Conception Rate; DCEG: Daughter Calving Ease; MTRG: Resistance to Metritis; RPRG: Resistance to Placental Retention. pts: Points; lbs: Pounds; %: Percentage; scr: score; NS: Not Significant; *r*: Pearson’s correlation coefficient; *p*: Probability.

## Supplementary Materials

The following are available online at https://www.mdpi.com/2076-2615/10/9/1704/s1, Table S1: Definition of genotypic/phenotypic parameters for production traits in high-producing primiparous dairy cows. Table S2: Definition of genotypic/phenotypic parameters for reproductive traits in high-producing primiparous dairy cows.
