# Supplementary material for: Genomic Evaluation of Primiparous High-Producing Dairy Cows: Inbreeding Effects on Genotypic and Phenotypic Production–Reproductive Traits
Source: Animals (Basel). 2020 Sep 21;10(9):1704. doi: 10.3390/ani10091704 (PMC7552765; doi:10.3390/ani10091704)
Supplement: Supplementary file 1 [file animals-10-01704-s001.zip › Table S1 Revised.pdf]

Table S1. Definition of genotypic/phenotypic parameters for production traits in high-producing primiparous cows (Supplementary Materials)

Traits and Indexes	Nomenclature	Units	Description	Parameter Type
Milk production at 305d	MP305G	lbs	Evaluates genetic differences in total pounds of milk produced during a 305-day lactation.	Genotype
Fat percentage at 305d	PF305G	%	Evaluates genetic differences in the percentage of milk fat produced during a 305-day lactation.	Genotype
Somatic Cell Count	SCCG	Score	Indirectly assesses susceptibility to mastitis. A low value indicates a more favourable value of somatic cells throughout lactation compared to the breed base.	Genotype
Milk Protein production at 305d	MPPROT305G	lbs	Evaluates genetic differences in pounds of milk-protein produced during a 305-day lactation.	Genotype
Protein percentage at 305d	PP305G	%	Evaluates genetic differences in percentage of milk-protein produced during a 305-day lactation.	Genotype
Milk fat production at 305d	MPFAT305G	lbs	Evaluates genetic differences in pounds of milk-fat produced during a 305-day lactation.	Genotype
Mastitis Resistance	MRG	%	Evaluates an animal's genetic resistance to mastitis. High values are consistent with animals with greater resistance to mastitis.	Genotype
Total Litres of Milk	TLM	L	Evaluates the total litres of milk produced during a 305-day lactation.	Phenotype
Total Fat	TFAT	lbs	Evaluates the amount of fat produced during a 305-day lactation.	Phenotype
Total Protein	TPROT	lbs	Evaluates the amount of protein produced during a 305-day lactation.	Phenotype
Milk projection at 305 d	MPJ305	L	Evaluates the amount of milk projected for a 305-day lactation.	Phenotype
Average Litres of milk	ALM	L	Evaluates the average milk in litres produced per day.	Phenotype
Fat Percentage	FATP	%	Evaluates the percentage of fat per litre of milk produced.	Phenotype
Protein Percentage	PROTP	%	Evaluates the percentage of protein per litre of milk produced.	Phenotype
Litres of Current Corrected Milk	LCCM	L	Evaluates the current amount of milk produced corrected to 305 days of lactation.	Phenotype
Milk Projection 305 days Maturity Equivalent	MPJ305ME	L	Estimates the projected milk production at 305 days which is equivalent to accumulated production maturity in each month.	Phenotype
Somatic Cell Count	SCC	$x10^3$/mL	Assesses the value of somatic cell concentration throughout lactation.	Phenotype
Logarithm of Somatic Cell Count	LGSCC	Log2-Score	Assesses the value of the adjusted somatic cell number in a lactation.	Phenotype
Urea	U	mg/dL	Evaluates a value that reflects the ureic nitrogen to a correct energy/protein balance.	Phenotype
