# Supplementary material for: Genomic Evaluation of Primiparous High-Producing Dairy Cows: Inbreeding Effects on Genotypic and Phenotypic Production–Reproductive Traits
Source: Animals (Basel). 2020 Sep 21;10(9):1704. doi: 10.3390/ani10091704 (PMC7552765; doi:10.3390/ani10091704)
Supplement: Supplementary file 1 [file animals-10-01704-s001.zip › Table S2 Revised.pdf]

Table S2. Definition of genomic/phenotypic parameters for reproductive traits in high-producing primiparous dairy cows (Supplementary Materials)

Traits and Indexes	Nomenclature	Units	Description	Parameter Type
Fertility Index	FIG	points	Estimates the reproductive efficiency in a female or a group of animals and incorporates several specific traits such as DPRG, CCRG, HCRG, DCEG, MTRG, RPRG.	Genotype
Heifer Conception Rate	HCRG	%	Estimates the increased percentage for the conception of the heifer, defined as the % of heifers inseminated that became pregnant in each service. HCR of 1 implies that the daughters of that heifer should be 1% more likely to become pregnant than the daughters of a heifer with a value of 0.	Genotype
Daughter's Pregnancy Rate	DPRG	%	Estimates the genetic ability of the animal for better reproductive efficiency and is expressed as the expected percentage difference, compared to the breed average, that an unpregnant cow will become pregnant during each 21-day estrous cycle. A 1.0 percent increase in DPRG is equivalent to a decrease of approximately four days open.	Genotype
Cow Conception Rate	CCRG	%	Estimates the genetic ability to conceive of the lactating cow defined as the percentage of inseminated cows that become pregnant in each service. A CCRG of 1 implies that daughters of that cow will be 1% more likely to become pregnant during lactation than daughters of a cow with a score of 0.	Genotype
Daughter's Calving Ease	DCEG	%	Estimates the genetic ability of a female to calve easily and is expressed as a percentage of dystocic deliveries for first calf-heifers. Lower numbers reflect easier calvings.	Genotype
Metritis Resistance	MTRG	%	Estimates the genetic resistance of heifers to metritis.	Genotype
Resistance to Placental Retention	RPRG	%	Estimates the genetic resistance of heifers to retained placenta.	Genotype
Weight at the First Service	WFS	kg	Evaluates the optimal weight of the heifer according to the breed at first service.	Phenotype
Number of Services per Conception: Nulliparous Cow	NSCNC	unit	Evaluates the average number of precise inseminations in a group of nulliparous cows to generate a pregnancy.	Phenotype
Days of Gestation	DG	days	Assesses the number of days the female remained pregnant until delivery.	Phenotype
Number of Services per Conception: Cow	NSCC	unit	Evaluates the average number of precise inseminations in a group of cows to generate a pregnancy. Informs about the fertility of the cows that were successful in reproduction.	Phenotype
Days in Milk at First Breeding	DMFB	days	Assesses the number of lactation days from birth to the first service.	Phenotype
Days in Anestrous Post-Voluntary Waiting Period	DAPVWP	days	Evaluates the number of post-partum days from voluntary waiting time to first insemination; values > 18 days determine anestrus.	Phenotype
Calving to Conception Interval	CCI	days	Assesses the time elapsed from birth to actual pregnancy.	Phenotype
Days Open Post Service	DOPS	days	Evaluates the period between the first insemination and the fertilizing insemination.	Phenotype
Efficiency in Heat Detection	EHD	%	Evaluates the average percentage in which oestrus is detected over a period of 21 days obtained by dividing 21 by the average interval between heats x 100 ((21 / AIHI) x 100).	Phenotype
Minimum Projected Inter-Partum Period	MPJIPP	days	Evaluates the time elapsed (as an adjusted projection) between one calving and another in the same cow.	Phenotype
Current Inter-Partum Period	CIPP	days	Evaluates the period between one calving and another in the same cow.	Phenotype
